# Combination Treatment with Cold Physical Plasma and Pulsed Electric Fields Augments ROS Production and Cytotoxicity in Lymphoma

**DOI:** 10.3390/cancers12040845

**Published:** 2020-03-31

**Authors:** Christina M. Wolff, Juergen F. Kolb, Klaus-Dieter Weltmann, Thomas von Woedtke, Sander Bekeschus

**Affiliations:** 1 ZIK Plasmatis, Leibniz Institute for Plasma Science and Technology (INP), a Member of the Leibniz Research Alliance Leibniz Health Technology, Felix-Hausdorff-Str. 2, 17489 Greifswald, Germany; christina.wolff@inp-greifswald.de (C.M.W.); weltmann@inp-greifswald.de (K.-D.W.); woedtke@inp-greifswald.de (T.v.W.); 2 Institute of Physics, University of Rostock, Albert-Einstein-Str. 23-24, 18059 Rostock, Germany; 3 Institute for Hygiene and Environmental Medicine, Greifswald University Medical Center, Ferdinand-Sauerbruch-Str., 17475 Greifswald, Germany

**Keywords:** PEF, plasma medicine, reactive oxygen and nitrogen species

## Abstract

New approaches in oncotherapy rely on the combination of different treatments to enhance the efficacy of established monotherapies. Pulsed electric fields (PEFs) are an established method (electrochemotherapy) for enhancing cellular drug uptake while cold physical plasma is an emerging and promising anticancer technology. This study aimed to combine both technologies to elucidate their cytotoxic potential as well as the underlying mechanisms of the effects observed. An electric field generator (0.9–1.0 kV/cm and 100-μs pulse duration) and an atmospheric pressure argon plasma jet were employed for the treatment of lymphoma cell lines as a model system. PEF but not plasma treatment induced cell membrane permeabilization. Additive cytotoxicity was observed for the metabolic activity and viability of the cells while the sequence of treatment in the combination played only a minor role. Intriguingly, a parallel combination was more effective compared to a 15-min pause between both treatment regimens. A combination effect was also found for lipid peroxidation; however, none could be observed in the cytosolic and mitochondrial reactive oxygen species (ROS) production. The supplementation with either antioxidant, a pan-caspase-inhibitor or a ferroptosis inhibitor, all partially rescued lymphoma cells from terminal cell death, which contributes to the mechanistic understanding of this combination treatment.

## 1. Introduction

Cold physical plasma applied in the field of plasma medicine is an ionized gas close to body temperature [1]. Plasmas are multi-component systems generating UV radiation, visible light, electromagnetic fields, thermal radiation, electrons and ions, and reactive oxygen species (ROS) and reactive nitrogen species (RNS) [2]. The latter play a crucial role in mediating the plasma effects in cells [3,4,5] by mediating oxidative modifications [6,7,8] to induce signaling pathways [9,10,11]. First discovered as a new tool for disinfection and wound healing [12,13,14], plasma also demonstrates cytotoxicity towards cancer cells [15,16,17]. A range of cellular responses have been found, including apoptosis, growth inhibition, cell cycle arrest, as well as cytoskeletal and mitochondrial damage [18,19,20]. The exact mechanism of plasma-induced apoptosis seems to be cell line dependent, varying from mitochondrial/intrinsic to receptor/extrinsic pathways as well as caspase- or Bcl-2 (B-cell lymphoma 2)-independent mechanisms [21,22,23].

Pulsed electric fields in the microsecond range (μsPEFs) are used in electrochemotherapy [24]. Application of µsPEF is an accredited anticancer therapy in the clinics [25]. µsPEFs transiently compromise the plasma cell membrane integrity of the cancer cells, allowing the chemotherapeutics, e.g., bleomycin and cisplatin, to enter the cell [26,27]. The cytotoxic effect is mainly achieved by the drugs [28]. Nevertheless, μsPEFs were also shown to perturb the membrane integrity of the endoplasmic reticulum (ER), causing an increase of the intracellular calcium level [29]. Therefore, PEFs with a higher pulse duration in the µs-range can, in principle, cause cell death via not only irreversible electroporation but also intracellular cell death signaling.

In our study, we aimed at combining the therapeutic principle of plasma treatment with that of PEF. Lymphoma suspension cell lines were used as a model system to investigate several cellular parameters after the combination treatment. We hypothesized that we would identify additive cytotoxicity because PEF-induced electropermeabilization may be enhanced by plasma leading to irreversible electroporation or because the combination of plasma and PEF results in an augmentation of ROS mediated intracellular cell death signaling.

## 2. Results

**Figure 1:**
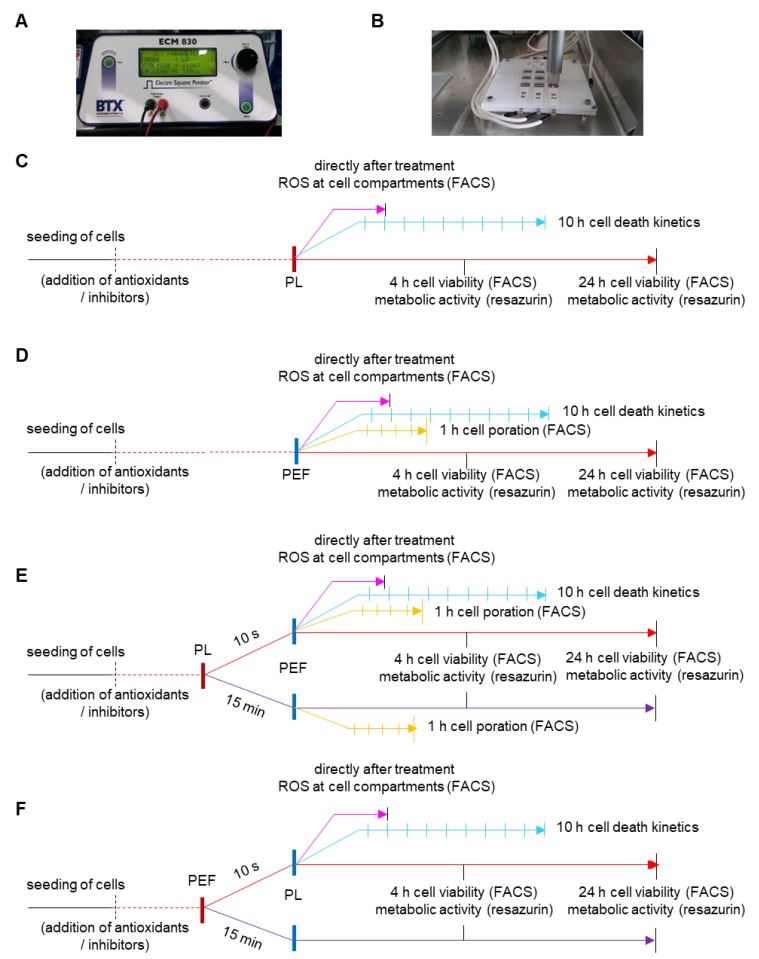
Experimental design. The electro square porator (**A**) was used for the application of pulsed electric fields. The cells were seeded in square Petri dishes, and a specially designed lid with electrodes allowed simultaneous treatment of plasma and PEF (**B**). A schematic overview of the mono treatments, plasma (**C**), and PEF (**D**), as well as the combination treatments, with first plasma and PEF second (**E**), and vice versa (**F**). PL = plasma; PEF = pulsed electric fields.

Several sequences were used for studying the combination treatment in comparison to the mono PEF (Figure 1C) and plasma (Figure 1D) treatment (Table 1). This included plasma treatment followed by PEF treatment and vice versa. In addition, the pause time between each of the treatment regimens was varied in some experiments to discriminate immediate from late effects (Figure 1E,F).

### 2.1. PEF but not Plasma Treatment Led to Cell Membrane Permeabilization

**Figure 2:**
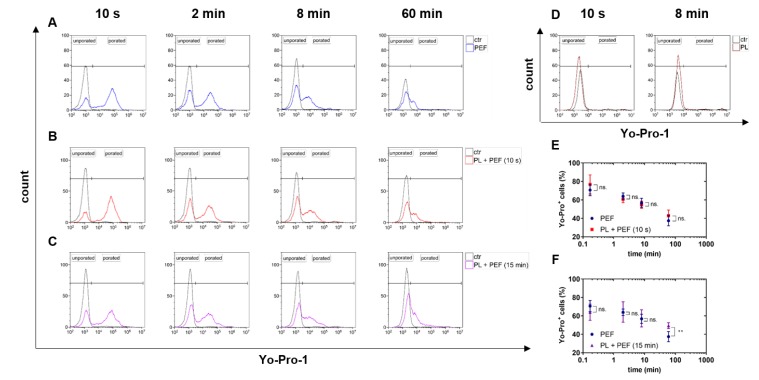
Electropermeabilization in PEF and plasma-treated lymphoma cells. The plasma exposure time was 90 s, and the electric field strength was 1 kV/cm. The electropermeabilization of cells was measured by the entry of Yo-Pro-1 10 s, 2 min, 8 min, and 60 min after the treatment using flow cytometry. The amount of electropermeabilized cells after PEF alone was administered (**A**) and was compared to cells pretreated with plasma 10 s before the PEF (**B**) or 15 min before the PEF (**C**). Plasma alone did not have any effect on the permeabilization of the cell membrane (**D**). Significant differences between the plasma-pretreated cells and the single PEF-treated cells were analyzed by t-test (**E**,**F**). Data are shown as one representative (**A**–**D**) or mean + S.E. (**E**,**F**) of at least four independent experiments. ctr = control; PL = plasma; PEF = pulsed electric fields; n.s. = not significant.

Pulsed electric fields (PEFs) are known to permeabilize the cellular plasma membrane. This so-called electropermeabilization was measured using the fluorescence dye Yo-Pro-1 and flow cytometry. The dye only enters cells with compromised but not intact membranes. The number of electropermeabilized cells was determined 10 s, 2 min, 8 min, and 60 min after the PEF treatment (Figure 2). The effect of plasma on the electropermeabilization due to its potential oxidation effect on the cell membrane was also validated. Regardless of the treatment regimen being PEF alone (Figure 2A), plasma plus PEF following immediately after each other (Figure 2B), or plasma plus PEF with 15 min in between both treatments (Figure 2C), the number of electropermeabilized cells decreased over time. Plasma treatment alone did not increase cell membrane permeability for Yo-Pro1 to enter (Figure 2D). If plasma treatment was immediately (10 s) followed by PEF treatment, similar results were obtained for both the overall decrease and the specific percentage of electropermeabilized cells (Figure 2E). When PEF treatment was performed 15 min after plasma treatment, a modest but significant increase in the percentage of permeabilized cells was observed after 1 h (Figure 2F). This suggests that pretreatment with plasma 15 min before the PEF treatment may cause more irreversible electroporation.

### 2.2. Combination of Plasma and PEF Treatment Showed Additive Toxicity

**Figure 3:**
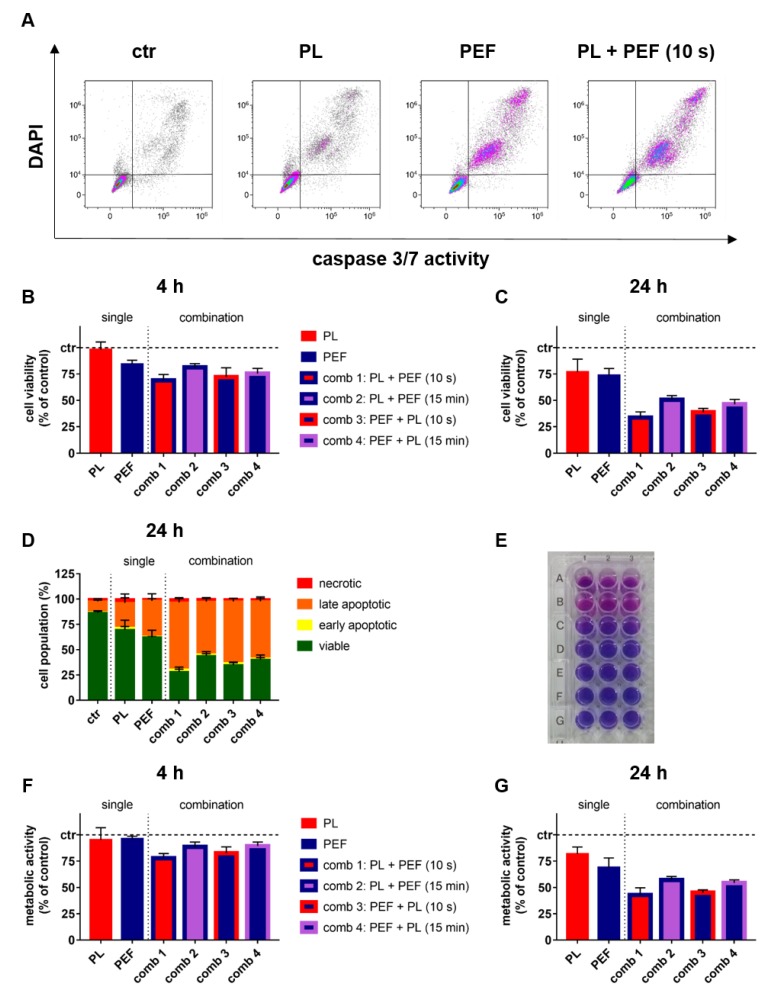
Cell viability and metabolic activity at 4 and 24 h after mono and combination treatments. The plasma exposure time was 40 s, and the electric field strength was 0.9 kV/cm. Percentages of viable, early, and late apoptotic, as well as necrotic cells were analyzed (**A**) and quantified (**B**–**D**) at 4 and 24 h using flow cytometry. The cell viability at 4 (**B**) and 24 h (**C**) is shown. The resazurin assay (**E**) was utilized to analyze the metabolic activity at 4 (**F**) and 24 h (**G**). The results of the t-test for the cell viability and metabolic activity are shown in Table 2 and Table 3, respectively. Data are shown as one representative (**A**,**E**) or mean + S.E. (**B**–**D**,**F**,**G**) of three independent experiments. ctr = control; PL = plasma; PEF = pulsed electric fields; comb = combination.

To understand the cytotoxic effects of the combination treatment, both cell viability and metabolic activity were determined at 4 and 24 h post-treatment. Compared to untreated control cells, both plasma and PEF treatment were cytotoxic at 24 h, with additive cytotoxicity observed for the combination treatment (Figure 3A). The quantitative analysis for the percentage of viable cells identified at 4 (Figure 3B) and 24 h (Figure 3C) confirmed this finding, indicating a statistically significant difference of the combination to the mono-treatments (Table 2). At 24 h, most of the dead cells were of a late-apoptotic phenotype (Figure 3D). Interestingly, not all combinations performed equally well. Exposure to plasma immediately following PEF was the most cytotoxic combination, with the vice versa sequence differing only to a minor extent. If there was a 15-min pause between either plasma and PEF or PEF and plasma treatment, the degree of cytotoxicity was reduced significantly (Table 2). Overall, similar findings were made (Table 3) when analyzing the metabolic activity (Figure 3E) of the lymphoma cells at 4 (Figure 3F) and 24 h (Figure 3G) after mono and combination treatment. These data suggested that combination treatment gave additive cytotoxic effects, while the sequence of combining plasma and PEF treatment was irrelevant as long as they were performed in close temporal proximity. Together with a lack of a substantial increase observed in the electroporated cells (Figure 2), this indicated that plasma and PEF enhance each other’s effect via intracellular signaling cascades rather than increased irreversible electroporation.

### 2.3. The Cell Death Kinetics of the Mono and Combination Treatments were Similar

**Figure 4:**
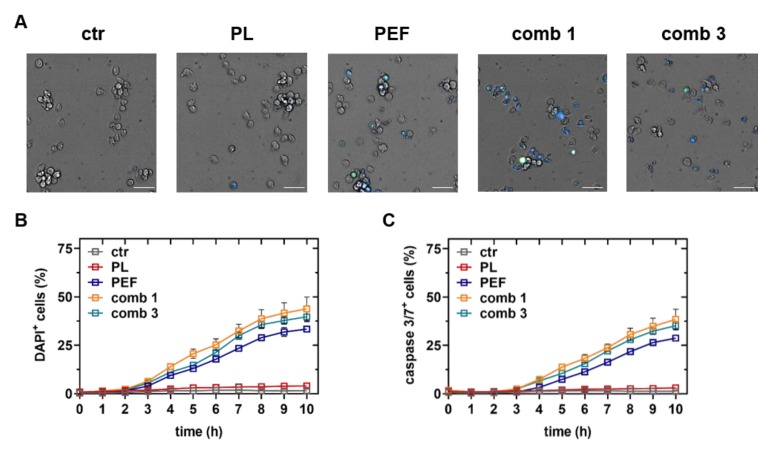
Cell death kinetics using live-cell imaging. The plasma-exposure time was 40 s, and the electric field strength was 0.9 kV/cm. Representative images of brightfield, caspase 3/7 (green), and 4′,6-diamidino-2-phenylindole (DAPI, blue) intensities 10 h after treatment of control, plasma, PEF, plasma plus PEF, and PEF plus plasma treatment (**A**). Quantitative analysis of DAPI (**B**) indicative of terminally dead cells and caspase 3/7 (**C**) indicative of apoptotic cells. Data are shown as one representative (**A**) and mean + S.E. (**B**,**C**) of three technical replicates from three independent experiments. ctr = control; PL = plasma; PEF = pulsed electric fields; comb = combination. Scale bar is 50 µm.

To investigate any potential differences of the cell death kinetics between the mono and combination treatments, apoptotic and terminally dead lymphoma cells (Figure 4A) were identified using algorithm-driven quantitative high content imaging microscopy. Plasma treatment did promote terminal (Figure 4B) or apoptotic (Figure 4C) cell death within a period of 10 h. These results were following our previous finding that plasma has no significant effect on the cell viability within the first four hours (Figure 3). By contrast, PEF treatment gave an increase in terminally dead cells (Figure 4B) and apoptotic cells (Figure 4C) starting at 3 and 4 h post-treatment, respectively. As observed when assaying metabolic activity and cell viability using flow cytometry (Figure 3), the combination treatment was more cytotoxic as compared to the PEF treatment alone. These results suggest that the plasma-induced cell death kinetic was slower than that of PEF. The earlier rise of the terminally dead cells as compared to the apoptotic cells in the PEF treatment regimens indicated that a portion of cell death was due to irreversible electroporation.

### 2.4. ROS and Lipid Peroxidation Contributed to Plasma and PEF Combination Treatment

**Figure 5:**
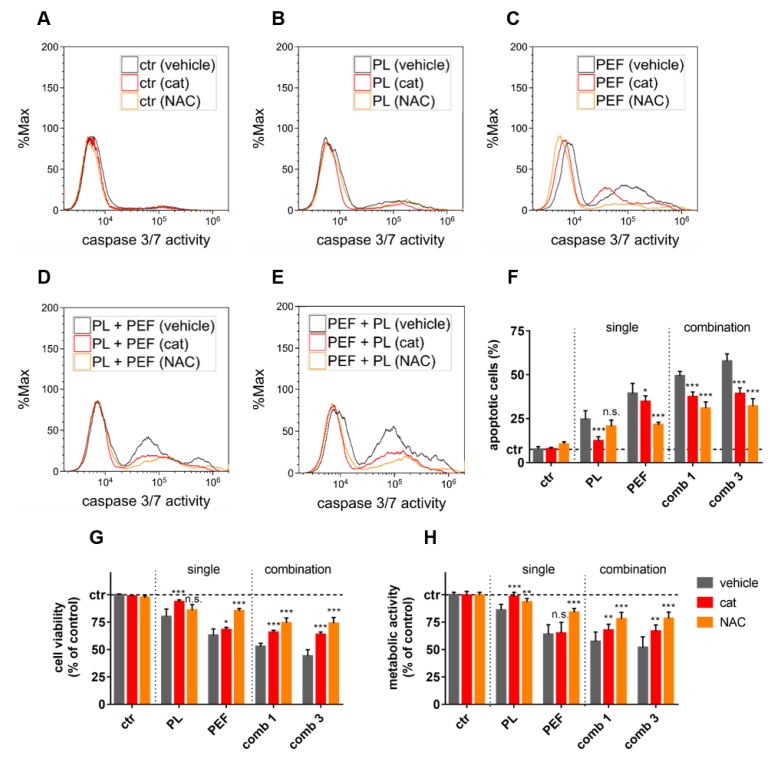
The effect of ROS scavenging in plasma and PEF treatments. The plasma exposure time was 40 s, and the electric field strength was 0.9 kV/cm. The cells were pretreated with catalase (cat) or N-acetylcysteine (NAC) and stained with CellEvent Caspase-3/7 Green Detection Reagent at 24 h in the control condition (**A**), or after plasma treatment (**B**), PEF treatment (**C**), plasma plus PEF treatment (**D**), or PEF plus plasma treatment (**E**). The apoptotic cells (caspase 3/7 active cells) were quantified 24 h after the treatments (**F**), as well as the cell viability (**G**) and the metabolic activity (**H**). The significant differences of cells without any antioxidants compared to the cat and NAC pretreated cells were determined by t-test. Data are shown as one representative (**A**–**E**) or mean + S.E. (**F**–**H**) of three independent experiments. ctr = control; PL = plasma; PEF = pulsed electric fields; comb = combination; n.s. = not significant.

To next decipher the role of ROS in cell death induction, the lymphoma cells were pretreated with catalase, an enzyme that detoxifies hydrogen peroxide (H_2_O_2_) [30], or N-acetylcysteine (NAC), a free radical scavenger and glutathione precursor [31] (Figure 5). While the antioxidants had no major effect on untreated lymphoma cells (Figure 5A), they decreased the number of caspase 3/7+ (apoptotic) cells after treatment with either plasma (Figure 5B), PEF (Figure 5C), plasma plus PEF (Figure 5D), or PEF plus plasma (Figure 5E) at 24 h post-treatment. The quantitative analysis revealed significant protection with both catalase and NAC from apoptosis (Figure 5F) and terminal cell death (Figure 5G) among the treatment regimens. This was confirmed when analyzing the total metabolic activity of the lymphoma cultures treated (Figure 5H). Catalase potently inhibited the cytotoxicity of plasma but barely affected PEF-treated cells. In the combined treatments, catalase was able to abolish the plasma effect. Conversely, NAC did not significantly suppress the plasma-induced cell death, but it was able to rescue a large amount of PEF-induced cell death. NAC also was more effective in inhibiting cytotoxicity in the combined treatment as compared to catalase. These findings suggest that ROS play a crucial role in the cell death signaling induced by both plasma and PEF.

**Figure 6:**
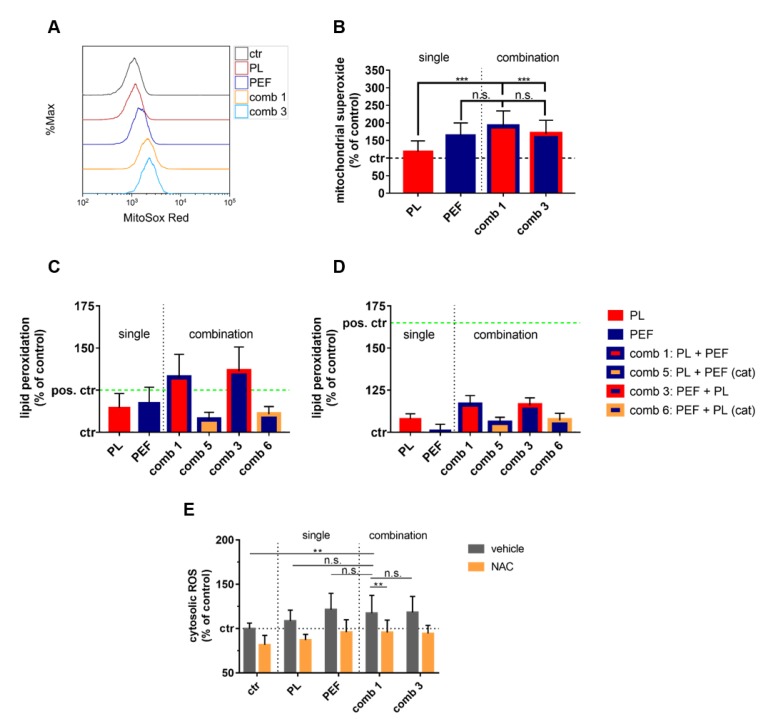
Cellular ROS and lipid peroxidation were observed with mono and combination treatments. The plasma exposure time was 40 s, and the electric field strength was 0.9 kV/cm. Mitochondrial superoxide was determined using MitoSox Red and flow cytometry (**A**) and quantified (**B**). A t-test was performed to determine significant differences. Lipid peroxidation was measured using boron-dipyrromethene (BODIPY) staining flow cytometry directly after treatment (**C**) and 1 h later (**D**). Cumene hydroperoxide (200 μM) was used as a positive control. Cytosolic ROS content was quantified using flow cytometry (**E**). The results of the t-test are shown in Table 4. Data are shown as one representative (**A**) or mean + S.E. (**B**–**E**) of three independent experiments. ctr = control; PL = plasma; PEF = pulsed electric fields; comb = combination; n.s. = not significant; cat = catalase.

While for plasma treatment ROS production has been extensively reported, this is less documented for µsPEF treatment. To analyze the potential contribution of endogenous ROS to the effects observed, mitochondrial superoxide generation was quantified immediately after the mono and combination treatment (Figure 6A). Intriguingly, PEF treatment led to potent superoxide production that was significantly increased when compared to that of plasma treatment alone (Figure 6B). As both endogenous and exogenous ROS are known to contribute to lipid peroxidation, its appearance was analyzed immediately (Figure 6C) and 1 h after mono and combination treatments (Figure 6D). A significant increase in lipid peroxidation was observed with all treatment regiments (Table 4), with the combination treatment and analysis immediately after the treatments showing the most substantial elevation. Pre-incubation with catalase significantly decreased lipid peroxidation. Furthermore, the lipid peroxidation was significantly higher in the combination than in the mono-treatments. No significant difference was observed between the combination treatment sequences. Altogether, we identified ROS scavenging to abrogate the plasma and PEF-induced cell death partially, which was concomitant with endogenous ROS production by mitochondria and lipid peroxidation. Moreover, an increased overall presence of ROS was found, which was significantly increased for the combination treatment as compared to control cells but not cells targeted with the single treatment modalities (Figure 6E).

### 2.5. Apoptosis and Ferroptosis Contributed to Plasma and PEF Combination Treatment

**Figure 7:**
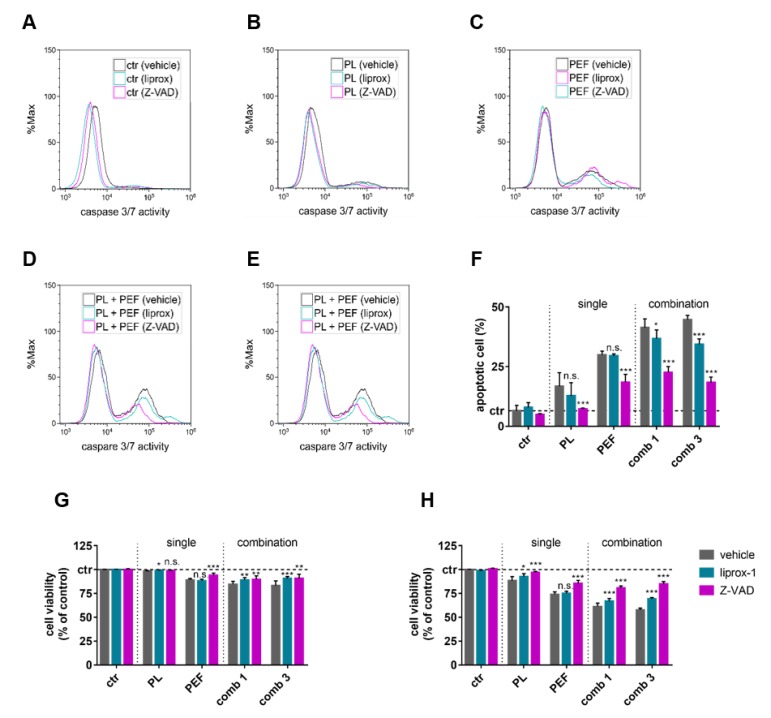
Influence of cell death inhibitors on cell viability after mono and combination treatments. The plasma exposure time was 40 s, and the electric field strength was 0.9 kV/cm. The cells were pretreated with liproxstatin-1 (liprox-1) or Z-VAD-FMK (Z-VAD) and stained with CellEvent Caspase-3/7 Green Detection Reagent 24 h after control conditions (**A**), plasma treatment (**B**), PEF treatment (**C**), plasma plus PEF treatment (**D**), or PEF plus plasma treatment (**E**). Apoptotic cells (caspase 3/7 active cells) were quantified 24 h after the treatments (**F**), as well as the cell viability after 4 (**G**) and 24 h (**H**). The significant differences of cells without any antioxidants compared to the liprox-1 and Z-VAD pretreated cells were determined by t-test. Data are shown as one representative (**A**–**E**) or mean + S.E. (**F**–**H**) of three independent experiments. ctr = control; PL = plasma; PEF = pulsed electric fields; comb = combination; n.s. = not significant.

Lipid peroxidation is a known constituent of the ferroptotic cell death signaling pathway, while apoptosis has been extensively described for both PEF and plasma-induced cell death. To test if the cytotoxicity of the mono and combination treatments was a consequence of signaling towards apoptosis or ferroptosis, the lymphoma cells were pre-incubated with either a pan-caspase inhibitor benzyloxycarbonyl-Val-Ala-Asp (OMe) fluoromethylketone (Z-VAD-FMK) or a ferroptosis inhibitor (liproxstatin-1). Both inhibitors had no effect in control cells (Figure 7A), while a reduction of the number of dead cells was observed in cells exposed to either plasma (Figure 7B), PEF (Figure 7C), plasma plus PEF (Figure 7D), or PEF plus plasma (Figure 7E). At 24 h post-treatment, the amount of caspase 3/7^+^ active cells was significantly decreased upon the addition of Z-VAD-FMK, regardless of the treatment regimen (Figure 7F), suggesting a critical role of apoptosis in the cell death pathways of all single and combined treatments. Liproxstatin-1 reduced the percentage of apoptotic cells in the combination of treatments. Similar trends were obtained when analyzing the percentage of viable cells at 4 h (Figure 7G) and especially at 24 h (Figure 7H) after the treatments. At 4 h, the extent of cell death was moderate, which is in line with the cell death kinetic data (Figure 4). In contrast to Z-VAD-FMK, the rescue effect of liproxstatin-1 was relatively small or absent, as in the case of PEF-treated cells. The most prominent cell death recovery achieved by liproxstatin-1 was obtained in cells exposed to PEF followed by plasma treatment (Figure 7F).

### 2.6. Validation of Additive Cytotoxicity in a Second Lymphoma Cell Line

**Figure 8:**
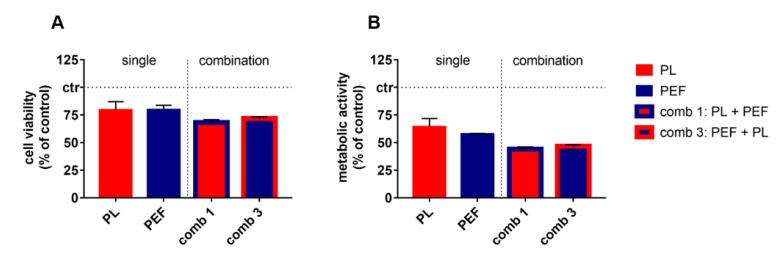
Cell viability and metabolic activity of U937 24 h after single and combination treatments. The plasma exposure time was 40 s, and the electric field strength was 1.25 kV/cm. The cell viability (**A**) was detected using CellEvent Caspase 3/7 Green Detection Reagent and sytox blue dead cell stain, and compared to the metabolic activity (**B**) determined using the resazurin assay. For a comprehensive overview, the statistical analysis is laid out below (Table 5). Data are shown as mean + S.E. of two independent experiments. PL = plasma; PEF = pulsed electric fields; comb = combination.

To validate the cytotoxic combination of plasma and PEF observed in Jurkat lymphoma cells, we analyzed the combination treatment in U937 cells to validate our findings (Figure 8). For the treatment of U937, a plasma exposure time of 40 s and an electric field strength of 1.25 kV/cm was used, based on pilot experiments identifying a modest but visible decrease in viability. After 24 h, the cytotoxic effects were studied by using the apoptosis-indicator dye CellEvent Caspase 3/7 and the terminal cell death indicator sytox blue. In parallel, the resazurin assay was employed to investigate metabolic activity at 24 h. The mono treatments decreased the viability of U937 to 75 % (Figure 8A). The combination treatments generated a significant additive cytotoxic effect when compared to the mono-treatments (Table 5). Similar results were obtained when analyzing the metabolic activity (Figure 8B), which also indicated significantly increased additive cytotoxicity of the combination treatment as compared to the mono-treatments. In contrast to the Jurkat cells, the decrease of U937 metabolic activity (Figure 8B) was much more prominent than that observed when analyzing cell viability (Figure 8A). Moreover, the combination effect, in general, was smaller in U937 as compared to Jurkat cells (Figure 3) but highly significant in both cases (Table 3 and Table 5).

## 3. Discussion

Oxidized membranes are known to be more easily electropermeabilized [32]. Therefore, we hypothesized that plasma-generated ROS enhance PEF-induced membrane electroporation and cytotoxicity [27,33]. Our second hypothesis was that the combination of plasma and PEF augments ROS production cell death signaling [29,34,35]. While three previous studies investigated the combination of plasma-treated liquids and PEF on cancer cells [36,37,38], our study aimed at elucidating the underlying mechanism of direct plasma treatment and pulsed electric fields (PEFs).

We found that plasma treatment followed by PEF exposure led to a modest increase in the irreversible electroporation of lymphoma cells. This corroborates previous findings using plasma-treated PBS [38]. In addition, our results showed a markedly increased cytotoxicity in the combination of the mono-treatments. This was more pronounced with simultaneous combination treatments as compared to a 15-min delay in between each of the physical treatment modalities. We can only speculate on the reasons responsible for this observation. One possibility might be that the simultaneous action of both PEF and plasma initiates and promotes cell death signaling pathways. By contrast, in the cell receiving PEF first and plasma 15 min later or vice versa, the stress response pathways initiated by the first treatment might have prepared the cells for a ‘second hit’ by increasing, for instance, antioxidant defense. With the minor increase in the number of electroporated cells with the combination treatment in mind, we hypothesized that intracellular signaling cascades enhanced the effects of single treatments rather than irreversible electroporation. Interestingly, the additive cytotoxicity of both treatment regimens was independent of the treatment sequence, suggestive of similar mechanisms at play. This seemed to be confirmed by our cell death kinetic experiments demonstrating a similar time course for the combined treatments. The slightly earlier signal increase of terminally dead over apoptotic cells for the PEF treatments indicates that part of the cells died independently of apoptosis. The fact that the combination treatment is significantly more toxic than the mono-treatments was confirmed in U937 cells in our study. U937 was selected due to the fact that the cell line was already used for comparison to Jurkat cells regarding their sensitivity against nanosecond pulsed electric fields [39,40,41]. Although the sensitivity of both cell lines towards the µsPEF and plasma used in our setup was similar, U937 were somewhat more robust towards the combination treatment-induced cell death. Reasons for this difference might be related to the expression of extrinsic apoptotic regulators as these were previously identified to cause different sensitivity towards nsPEF [39].

To generate a deeper understanding of the underlying mechanism, the role of ROS was investigated in the mono and combination treatments. The potent rescue with catalase for plasma-induced apoptosis revealed H_2_O_2_ as the primary mediator of plasma-induced cell death, as previously suggested [42,43]. Consequently, the plasma effect of the combined treatment was abolished by catalase. The general antioxidant NAC was also able to abrogate the cytotoxicity of PEF partially. Apparently, ROS play an important role in the cell death mechanism for not only plasma but also PEF treatment. The scavenging of ROS might interfere with pro-apoptotic signaling as well as direct inhibition of mitochondria-derived ROS, triggering apoptosis [44]. Likewise, NAC rescued cells exposed to combination treatments from apoptosis, demonstrating to be a more potent inhibitor than catalase.

Further evidence for the critical role of ROS in the mono and combination treatments was found from the increase in lipid peroxidation and mitochondrial superoxide release observed. Both lipid peroxidation and mitochondrial ROS were significantly increased in the combination over the mono-treatments. In contrast to the PEF-treated cells, the lipid peroxidation was still measurable after one hour in plasma and combined-treated cells. It is worth mentioning that the accumulation of lipid peroxidation is one characteristic of ferroptosis [45]. Another characteristic is lethal ROS derived from iron metabolism [46]. Ferroptosis was previously discussed as a cell death mechanism for plasma [47]. The combination of plasma and PEF might enhance this form of regulated cell death that is also intertwined with mitochondria and ROS release [48]. To investigate the role of mitochondria in the mono and combination treatments, the mitochondria superoxide release was measured. In general, the mitochondrial membrane permeability increases during apoptosis, releasing pro-apoptotic factors into the cytosol. Furthermore, ROS generated by mitochondria can also be involved in cell death [44]. A substantial increase of mitochondrial superoxide release measured for PEF and combination treatment strongly suggests an involvement of mitochondria in the cell death mechanism. It might also explain the rescue effect of NAC for these treatments.

A rescue from apoptosis of the pan-caspase inhibitor Z-VAD-FMK and the ferroptosis inhibitor liproxstatin-1 was identified. Z-VAD-FMK was able to significantly reduce the cytotoxicity of all treatments, showing that apoptosis is one primary cell death mechanism. Nevertheless, a substantial number of cells were still dying after the PEF and combination treatments, indicating more than one cell death mechanism. One potential candidate is ferroptosis. Indeed, liproxstatin-1 increased the cell viability of plasma and combination treatments, suggesting ferroptosis as another cell death mechanism. However, the cytotoxicity of PEF seemed to be independent of ferroptosis. Interestingly, the rescue effect of liproxstatin-1 was more prominent for the cells treated first with PEF followed by plasma exposure. The fact that liproxstatin-1 also reduced caspase 3/7 activity, a marker for apoptosis [45], could be explained by ferroptosis-induced apoptosis. It was previously shown that erastin, a ferroptosis inducer, can promote TRAIL (tumor necrosis factor related apoptosis-inducing ligand)-induced apoptosis [49]. Similar crosstalk might have occurred in our experiments. It is also possible that liproxstatin-1 directly inhibits apoptosis while suppressing lipid peroxidation [50] since the end products of lipid peroxidation can also cause apoptosis [51].

Future studies using, for instance, more realistic models in cancer research, such as 3D tumor spheroids and tumors growing in vivo, may shed more light on the clinical relevance of our findings. In addition, the usage of adherent cancer cell lines as targeted during ECT might have been more relevant from the clinical perspective. However, our study mainly aimed to identify the mechanism of action of the two physical treatment regimens PEF and plasma, and not necessarily the clinical applicability of such an approach. Moreover, lymphomas are still cancer cells that share several driver mutations with those found in solid tumors. Nevertheless, there are challenges with the applicability of the concept of using PEF and plasma in clinical therapy, which we have outlined previously [25]. Among those are, for example, in melanoma, the location of many metastases beneath the skin in the stratum corneum, which are difficult to reach directly with plasmas. Subsequently, an ideal combination therapy would require a device performing electroporation and plasma-delivery at the same time. Regarding the next steps in plasma cancer treatment, we feel it might be important to continue combining plasma treatment with existing treatment regimens in oncology. This way, plasma treatment might become an adjuvant in addition to clinically accredited procedures.

## 4. Material and Methods

### 4.1. Cell Culture

The lymphoma cell lines Jurkat (ACC282) and U-937 (ACC5) were used. The cells were cultured in Roswell Park Memorial Institute (RPMI) 1640 medium (Corning, Wiesbaden, Germany) containing 10% fetal bovine serum (Sigma-Aldrich, Taufkirchen, Germany), 1% glutamine (Corning, Wiesbaden, Germany), and 1% penicillin/streptomycin (Corning, Wiesbaden, Germany) at 37 °C and 5% CO_2_. At 7.5 × 10^5^ per 1.5 mL of medium, the cells were seeded into square Petri dishes having 25 compartments, each with a surface area of 3.7 cm² (Thermo Scientific, Zurich, Switzerland), directly before the treatment. Catalase (cat, 20 µg/mL; Sigma-Aldrich, Taufkirchen, Germany) or N-acetylcysteine (NAC, 2 mM; Sigma-Aldrich, Taufkirchen, Germany) were used as the ROS scavenger. The following inhibitors were used to interfere with apoptosis or ferroptosis signaling pathways: Z-VAD-FMK (50 µM; AdooQ Bioscience, Irvine, CA, USA), a pan-caspase inhibitor, and liproxstatin-1 (50 nM; Sigma-Aldrich, Taufkirchen, Germany), an inhibitor of lipid peroxidation [50].

### 4.2. Exposure to Cold Physical Plasma and Pulsed Electric Fields

As a plasma source, an atmospheric pressure plasma jet (kINPen [52]) was utilized. The plasma jet was operated at two standard liters per minute using argon gas. Exposure times per well were 40 or 90 s, depending on the experiments. The ideal plasma treatment times were identified in pilot experiments to have a modest but visible decline in cell viability. The treatment time likely depends on our specific setup and may be different for other types of plasma sources. In all approaches, the cells were treated directly. For the application of pulsed electric fields (PEFs), an electro square porator (ECM 830; BTX Harvard Apparatus, Holliston, MA, USA) was used (Figure 1A). The PEF had a pulse duration of 100 μs and an electric field strength of 1, 0.9, or 1.25 kV/cm depending on the type of experiment; they were applied as eight consecutive pulses at 1 Hz. To treat the cells directly in the square Petri dishes, a lid with plate electrodes was designed, allowing individual control of the compartments (Figure 1B) for all experimental procedures performed (Figure 1C–F).

### 4.3. Analysis of Electropermeabilization

To test for electropermeabilization, the entry of the green fluorescent dye Yo-Pro-1 (Thermo Fisher Scientific, Dreieich, Germany) was determined via flow cytometry (CytoFLEX S; Beckman-Coulter, Brea, CA, USA). PEF of eight consecutive pulses with an electric field strength of 1 kV/cm and a pulse duration of 100 µs was applied. For the plasma treatment, the cells were exposed to 90 s of Argon-plasma. The plasma treatment was done either directly (10 s) or 15 min before the PEF treatment. Yo-Pro-1 (final concentration 0.5 µM) was added to the cells for 15 min at a pre-determined frequency (10 s, 2 min, 8 min, and 1 h after the mono and combination treatments). Subsequently, the Yo-Pro-1 mean fluorescence intensity of each cell was measured using flow cytometry. Sample analysis was performed utilizing Kaluza 2.1 software (Beckman-Coulter, Brea, CA, USA).

### 4.4. Metabolic Activity and Cell Viability

The metabolic activity was determined by the conversion of resazurin (final concentration 100 µM) to resorufin [53]. The cells were incubated with resazurin (Alfa Aesar, Haverhill, MA, USA) before quantification of the fluorescent product resorufin at 590 nm (excitation wavelength 535 nm) using a microplate reader (F200; Tecan, Männedorf, Switzerland). The absolute fluorescent intensities of the samples were normalized to that retrieved from the untreated control cells. For the analysis of apoptotic and terminally dead cells, either 4′,6-diamidino-2-phenylindole (DAPI, 1 µM; Sigma-Aldrich, Taufkirchen, Germany) or Sytox Blue (1 µM; Thermo Fisher Scientific, Dreieich, Germany) and CellEvent Caspase-3/7 Green Detection Reagent (1 µM; Thermo Fisher Scientific, Dreieich, Germany) was utilized. After 15 min of incubation at 37 °C, the percentage of each cell population staining negative or single or double-positive for the dyes was discriminated using flow cytometry.

### 4.5. Cell Death Kinetics Using Live-Cell Microscopy

For time-lapse imaging experiments, DAPI (1 µM) and Caspase-3/7 Green Detection Reagent (1.2 µM) was added to detect apoptotic and terminally dead cells. The cells were incubated in a high content imaging system (Operetta CLS; PerkinElmer, Hamburg, Germany) at 37 °C and 5% CO_2_ for 10 h. For each sample, several technical replicates were added to the 96-well plates. For each well, 9 fields of view were imaged at a frequency of 1 h^−1^ using an air 20× air objective (NA = 0.16; Zeiss) to capture the brightfield, digital phase contrast, and fluorescence channels for Caspase-3/7 Green Detection Reagent (λ_ex_ 475nm and λ_em_ 500–550 nm) and DAPI (λ_ex_ 365 nm and λ_em_ 430–500 nm). A sCMOS camera (4.7 MP) acquired the images at 16 bit. The rim of the 96-well plates was filled was sterile water for evaporation protection. Image quantification was performed using Harmony 4.9 software (PerkinElmer, Hamburg, Germany).

### 4.6. Oxidation of Cell Membrane and Mitochondria

To assess oxidation of the cell membrane and mitochondria, the cells were stained with DAPI and BODIPY 581/591 C11 (1 µM; Thermo Fisher Scientific, Dreieich, Germany) or MitoSox Red Mitochondrial Superoxide Indicator (1 µM; Thermo Fisher Scientific, Dreieich, Germany) in phosphate-buffered saline (PBS) for 15 min. Afterward, the cells were washed, resuspended in fully supplemented RPMI medium, and treated as described above. Immediately after, the samples were acquired using flow cytometry. Cumene hydroperoxide (200 μM; Sigma-Aldrich, Taufkirchen, Germany) was used as a positive control for lipid peroxidation. Cytosolic ROS content was quantified using CM-H_2_-DCF-DA (1 µM; Thermo Fisher Scientific, Dreieich, Germany) as per the manufacturer’s instructions and quantified utilizing flow cytometry.

### 4.7. Statistical Analysis

For each assay, if not indicated otherwise, at least three independent experiments, each with at least three technical replicates, were performed and included in the data analysis. Data were analyzed and graphed using prism 8.4 (GraphPad Software, San Diego, CA, USA). Multiple t-test analysis with post-hoc testing was employed to determine the degree of statistical significance found between the values of the different groups. The level of significance is indicated as follows: *p* < 0.05 (*), *p* < 0.01 (**), and *p* < 0.001 (***).

## 5. Conclusions

Our first hypothesis that plasma treatment oxidizes cellular membranes and, by this, sensitizes cells to increased PEF-induced electroporation was not confirmed. By contrast, we confirmed our second hypothesis that plasma and PEF treatment synergize to augment ROS production, resulting in additive cytotoxicity. Moreover, we found that the combination treatment is most potent when performed simultaneously instead of sequentially with 15 min in between each treatment. Lastly, we identified not one but several mechanisms at play contributing to the combination treatment-mediated cytotoxic responses. This included caspase-dependent apoptosis, ROS from exogenous and endogenous compartments, ferroptosis, and PEF-induced necrosis.

## Figures and Tables

**Table 1:** Explanation of the abbreviations for the combination (comb) treatment regimens.

Abbreviation	Combination Treatment
comb 1	combination of first plasma and second PEF waiting for 10 s in between
comb 2	combination of first plasma and second PEF waiting 15 min in between
comb 3	combination of first PEF and second plasma waiting for 10 s in between
comb 4	combination of first PEF and second plasma waiting 15 min in between
comb 5	pretreatment with catalase before combination of first plasma and second PEF waiting for 10 s in between
comb 6	pretreatment with catalase before combination of first PEF and second plasma waiting for 10 s in between

**Table 2:** Statistical analysis of cell viability 4 and 24 h after treatment. A multiple t-test was performed (* *p* < 0.05; ** *p* < 0.01; and *** *p* < 0.001). ctr = control; PL = plasma; PEF = pulsed electric fields; comb = combination; n.s. = not significant.

*Treatment Regimen*	4 h				24 h			
	Significance	*p*-Value	Difference	S.E.	Significance	*p*-Value	Difference	S.E.
ctr / PL	n.s.	0.400	1.82	2.14	***	<0.001	23.01	3.53
ctr / PEF	***	<0.001	15.66	1.17	***	<0.001	26.29	1.90
ctr / comb 1	***	<0.001	31.03	1.67	***	<0.001	66.23	1.55
ctr / comb 3	***	<0.001	27.88	2.59	***	<0.001	61.37	1.10
ctr / comb 2	***	<0.001	18.66	1.14	***	<0.001	49.45	1.19
ctr / comb 4	***	<0.001	24.55	1.51	***	<0.001	53.75	1.36
PL / PEF	***	<0.001	13.84	2.35	n.s.	0.420	3.29	3.99
PL / comb 1	***	<0.001	29.21	2.64	***	<0.001	43.22	3.84
PL / comb 3	***	<0.001	26.05	3.30	***	<0.001	38.36	3.68
PL / comb 2	***	<0.001	16.83	2.63	***	<0.001	26.44	4.25
PL / comb 4	***	<0.001	22.72	2.81	***	<0.001	30.74	4.30
PEF / comb 1	***	<0.001	15.37	1.93	***	<0.001	39.94	2.42
PEF / comb 3	***	<0.001	12.21	2.77	***	<0.001	35.07	2.16
PEF / comb 2	n.s.	0.080	3.00	1.61	***	<0.001	23.16	2.46
PEF / comb 4	***	<0.001	8.89	1.89	***	<0.001	27.45	2.55
comb 1 / comb 3	n.s.	0.310	−3.157	3.02	*	0.020	−4.87	1.86
comb 1 / comb 2	***	<0.001	−12.38	2.12	***	<0.001	−16.78	2.11
comb 1 / comb 4	*	0.010	−6.49	2.34	***	<0.001	−12.48	2.21
comb 3 / comb 2	**	0.008	−9.22	3.13	***	<0.001	−11.91	1.68
comb 3 / comb 4	n.s.	0.320	−3.33	3.29	***	<0.001	−7.62	1.81
comb 2 / comb 4	*	0.010	5.89	2.02	n.s.	0.050	4.30	2.04

**Table 3:** Statistical analysis of metabolic activity 4 and 24 h after treatment. A multiple t-test was performed (* *p* < 0.05; ** *p* < 0.01; and *** *p* < 0.001). ctr = control; PL = plasma; PEF = pulsed electric fields; comb = combination; n.s. = not significant.

*Treatment Regimen*	4 h				24 h			
	Significance	*p*-Value	Difference	S.E.	Significance	*p*-Value	Difference	S.E.
ctr / PL	n.s.	0.190	4.72	3.52	***	<0.001	18.12	2.01
ctr / PEF	***	<0.001	3.80	0.89	***	<0.001	30.75	2.63
ctr / comb 1	***	<0.001	22.15	1.42	***	<0.001	57.09	2.08
ctr / comb 3	***	<0.001	17.50	1.89	***	<0.001	54.99	1.00
ctr / comb 2	***	<0.001	11.46	1.49	***	<0.001	42.87	1.23
ctr / comb 4	***	<0.001	10.54	1.22	***	<0.001	45.62	1.12
PL / PEF	n.s.	0.790	−0.92	3.42	***	<0.001	12.63	3.28
PL / comb 1	***	<0.001	17.43	3.58	***	<0.001	38.96	2.82
PL / comb 3	**	0.003	12.78	3.77	***	<0.001	36.87	2.07
PL / comb 2	n.s.	0.120	6.74	4.10	***	<0.001	24.75	2.44
PL / comb 4	n.s.	0.160	5.82	4.02	***	<0.001	27.50	2.38
PEF / comb 1	***	<0.001	18.35	1.48	***	<0.001	26.33	3.23
PEF / comb 3	***	<0.001	13.70	1.90	***	<0.001	24.24	2.67
PEF / comb 2	***	<0.001	7.66	1.58	**	0.001	12.12	3.13
PEF / comb 4	***	<0.001	6.45	1.36	***	<0.001	14.87	3.08
comb 1 / comb 3	*	0.040	−4.65	2.18	n.s.	0.340	−2.10	2.14
comb 1 / comb 2	***	<0.001	−10.69	2.00	***	<0.001	−14.21	2.51
comb 1 / comb 4	***	<0.001	−11.61	1.83	***	<0.001	−11.46	2.46
comb 3 / comb 2	*	0.020	−6.04	2.44	***	<0.001	−12.12	1.34
comb 3 / comb 4	**	0.007	−6.96	2.30	***	<0.001	−9.37	1.24
comb 2 / comb 4	n.s.	0.650	−0.92	1.98	n.s.	0.090	2.75	1.52

**Table 4:** Statistical analysis of lipid peroxidation immediately and 1 h after treatment. A multiple t-test was performed (* *p* < 0.05; ** *p* < 0.01; and *** *p* < 0.001). ctr = control; PL = plasma; PEF = pulsed electric fields; comb = combination; cat = catalase; n.s. = not significant.

*Treatment Regimen*	Immediately after Treatment				1 h after Treatment			
	Significance	*p*-Value	Difference	S.E.	Significance	*p*-Value	Difference	S.E.
ctr / PL	***	<0.001	−14.52	2.22	***	<0.001	−7.86	0.93
ctr / PEF	***	<0.001	−17.32	2.46	n.s.	0.300	−1.12	1.05
ctr / comb 1	***	<0.001	−32.59	3.56	***	<0.001	−16.30	1.49
ctr / comb 1 + cat	***	<0.001	−7.58	1.21	***	<0.001	−5.62	1.03
ctr / comb 3	***	<0.001	−36.21	3.76	***	<0.001	−15.95	1.24
ctr / comb 3 + cat	***	<0.001	−10.65	1.29	***	<0.001	−7.01	1.25
PL / PEF	n.s.	0.400	−2.80	3.26	***	<0.001	6.74	1.26
PL / comb 1	***	<0.001	−18.07	4.15	***	<0.001	−8.44	1.63
PL / comb 1 + cat	*	0.030	6.94	3.04	n.s.	0.110	2.24	1.35
PL / comb 3	***	<0.001	−21.69	4.32	***	<0.001	−8.09	1.41
PL / comb 3 + cat	n.s.	0.220	3.87	3.07	n.s.	0.580	0.85	1.52
PEF / comb 1	**	0.001	−15.28	4.28	***	<0.001	−15.18	1.74
PEF / comb 1 + cat	**	0.008	9.74	3.33	**s	0.007	−4.50	1.50
PEF / comb 3	***	<0.001	−18.90	4.45	***	<0.001	−14.83	1.52
PEF / comb 3 + cat	n.s.	0.060	6.67	3.37	**	0.002	−5.89	1.66
comb 1 / comb 1 + cat	***	<0.001	25.01	4.73	***	<0.001	10.68	2.03
comb 1 / comb 3	n.s.	0.490	−3.62	5.14	n.s.	0.850	0.349	1.83
comb 1 / comb 3 + cat	***	<0.001	4.62	4.62	***	<0.001	9.29	2.14
comb 1 + cat / comb 3	***	<0.001	−28.63	4.98	***	<0.001	−10.33	1.72
comb 1 + cat / comb 3 + cat	n.s.	0.160	−3.07	2.09	n.s.	0.450	−1.39	1.81
comb 3 / comb 3 + cat	***	<0.001	25.56	5.00	***	<0.001	8.94	1.86

**Table 5:** Statistical analysis of cell viability and metabolic activity 24 h after treatment. A multiple t-test was performed (* *p* < 0.05; ** *p* < 0.01; and *** *p* < 0.001). ctr = control; PL = plasma; PEF = pulsed electric fields; comb = combination; n.s. = not significant.

*Treatment Regimen*	Cell Viability				Metabolic Activity			
	Significance	*p*-Value	Difference	S.E.	Significance	*p*-Value	Difference	S.E.
ctr / PL	***	<0.001	20.50	3.09	***	<0.001	35.54	3.04
ctr / PEF	***	<0.001	20.15	1.60	***	<0.001	42.24	0.34
ctr / comb 1	***	<0.001	31.77	1.08	***	<0.001	55.98	0.90
ctr / comb 3	***	<0.001	28.14	0.61	***	<0.001	53.31	0.63
PL / PEF	n.s.	0.920	0.35	3.47	n.s.	0.050	−6.69	3.03
PL / comb 1	**	0.006	−11.27	3.27	***	<0.001	−20.43	3.14
PL / comb 3	n.s.	0.050	−7.64	3.46	***	<0.001	−17.76	3.08
PEF / comb 1	***	<0.001	11.62	1.92	***	<0.001	13.74	0.88
PEF / comb 3	**	0.002	7.99	1.86	***	<0.001	11.07	0.59
comb 1 / comb 3	*	0.020	−3.63	1.33	*	0.020	−2.67	1.02
